# Modeling and Analysis of Neural Spike Trains

**DOI:** 10.1155/2014/161203

**Published:** 2014-07-03

**Authors:** Wei Wu, Asohan Amarasingham, Zhe (Sage) Chen, Sung-Phil Kim

**Affiliations:** ^1^Department of Statistics, Florida State University, Tallahassee, FL 32306, USA; ^2^Department of Mathematics, The City College of New York, New York, NY 10031, USA; ^3^Department of Psychiatry and Department of Neuroscience and Physiology, New York University School of Medicine, New York, NY 10016, USA; ^4^School of Design and Human Engineering, Ulsan National Institute of Science and Technology, UNIST-gil 50, Ulsan 689-798, Republic of Korea

It is well known that time-dependent information is represented via sequences of stereotyped spike waveforms in the nervous system. Mathematical modeling and analysis of waveform sequences (or spike trains) have been one of the central problems in the field of computational neuroscience. This problem is significantly challenging since population neuronal activity is often stochastic, highly correlated, and nonstationary across time. A great deal of effort has been devoted to characterizing this activity by using state-of-the-art methodologies, such as artificial neural networks, signal processing methods, adaptive filtering theory, parametric and nonparametric probabilistic models, Bayesian inference, metric-based analysis, and information-theoretic methods. Advances in technology have enabled us to record larger-scale neuronal ensemble activity, and current research has devoted a lot to integrating and analyzing increasingly large-volume, high-dimensional, and fine-grain experimental data.

The main focus of this special issue is to provide an international forum for researchers to present the most recent developments and innovative ideas in the field. We aim to incorporate new contributions in theories, algorithms, and applications. A total of 17 submissions, which cover a broad spectrum of spike train modeling and analysis, were received for this special issue. Each submission was rigorously reviewed by external referees as well as the Guest Editor. To ensure the high quality of papers, we finally accepted 6 articles for this special issue. The following is a brief summary for each of these accepted articles.

The paper “*An overview of Bayesian methods for neural spike train analysis*” by Z. Chen presents a tutorial overview of Bayesian methods and their representative applications in neural spike train analysis, at both single neuron and population levels. On the theoretical side, the focus is on various approximate Bayesian inference techniques as applied to latent state and parameter estimation. On the application side, the topics include spike sorting, tuning curve estimation, neural encoding and decoding, deconvolution of spike trains from calcium imaging signals, and inference of neuronal functional connectivity and synchrony.

The paper “*A tensor-product-kernel framework for multiscale neural activity decoding and control*” by L. Li et al. proposes a tensor-product-kernel framework for integrating multiscale neural activity and applies it to an offline stimulus decoding and an open-loop control task in brain-machine interface. Choosing the kernels is equivalent to identifying a proper spatiotemporal scale among neural activity such as spike trains and local field potentials. This work provides a general framework to leverage heterogeneous neural activities recorded from neuroscience experiments.

The paper “*Homogenous chaotic network serving as a rate/population code to temporal code converter*” by M. V. Kiselev addresses an important relationship between rate coding and temporal coding in neuroscience and shows that conversation from the rate code to temporal code can be implemented by a homogeneous chaotic neural network. The paper demonstrates this approach using simulated leaky integrate-and-fire neurons and spiking network in line with the selective polychromous neuronal groups. It shows that the quality of conversation is dependent on a wide range of parameters, such as the stimulus diversity, intensity, background noise, and excitatory connection delays.

The paper “*Prediction of human's ability in sound localization based on the statistical properties of spike trains along the brainstem auditory pathway*” by R. Krips and M. Furst demonstrates that aspects of the human ability to perform auditory localization are compatible with a theory of optimal estimation applied to responses of the auditory nerve and superior olivary complex. The nature of this fit is suggestive of the sources of information used to perform this computation.

The paper “*Spike sorting by joint probabilistic modeling of neural spike trains and waveforms*” by B. A. Matthews and M. A. Clements develops a novel probabilistic method for automatic neural spike sorting which uses stochastic point process models of neural spike trains and parameterized action potential waveforms. A novel likelihood model for observed firing times as the aggregation of hidden neural spike trains is derived as well as an iterative procedure for clustering the data and finding the parameters that maximize the likelihood.

The paper “*Sparse data analysis strategy for neural spike classification*” by V. Vigneron and H. Chen deals with the problem of identifying single neuronal units from multichannel extracellular recordings. This study proposes a family of metrics based on different levels of parsimony to separate spike waveform data into single unit waveforms. It demonstrates that the proposed method can provide an effective spike-sorting tool to visually analyze the spike data and to produce robust results for noisy, imbalanced, and highly correlated spike waveform data.

In summary, these six papers offer many examples of active research topics in neural spike train analysis. We thank all authors for submitting their papers to this special issue. We also thank all reviewers for providing their expertise and making valuable comments during the reviewing process. It is our hope that these papers would provide novel ideas and methodological development to the neural spike train modeling community, and the results would be useful for better understanding of neuronal mechanisms in the brain and providing more effective applications.

